# Effectiveness of a virtual reality-based interventional radiology simulator for medical student education

**DOI:** 10.1007/s11604-025-01771-z

**Published:** 2025-03-29

**Authors:** Hidenori Mitani, Yukiko Honda, Keigo Narita, Yuko Nakamura, Shintaro Morishita, Shota Kondo, Shogo Maeda, Haruka Higashibori, Keigo Chosa, Toru Higaki, Ikuo Kawashita, Minoru Hattori, Naoko Hasunuma, Isamu Saeki, Shinya Takahashi, Naoki Mihara, Kazuo Awai

**Affiliations:** 1 Department of Diagnostic Radiology, Graduate School of Biomedical and Health Sciences, Hiroshima University, 1-2-3 Kasumi, Minami-ku, Hiroshima, 7340037 Japan; 2 Visual Information Science Laboratory, Graduate School of Advanced Science and Engineering, Hiroshima University, 1-4-1 Kagamiyama, Higashi-Hiroshima City, Hiroshima 739-8527 Japan; 3 Center for Medical Education, Hiroshima University, 1-2-3 Kasumi, Minami-ku, Hiroshima, 7340037 Japan; 4 Department of Pediatric Surgery, Hiroshima University Hospital, 1-2-3 Kasumi, Minami-ku, Hiroshima, 7340037 Japan; 5 Department of Cardiovascular Surgery, Graduate School of Biomedical and Health Sciences, Hiroshima University, 1-2-3 Kasumi, Minami-ku, Hiroshima, 7340037 Japan; 6 Department of Diagnostic Radiology, Iseikai International General Hospital, 4-14 Minamiougi-Machi, Kita-ku, Osaka, 5300052 Japan

**Keywords:** Medical student education, Interventional radiology, Virtual reality, Interventional radiology simulator, Augmented reality

## Abstract

**Purpose:**

We developed an interventional radiology (IR) simulator using a virtual reality system (the VR–IR simulator) to teach IR procedures to medical students. In this study, we investigated the effectiveness of this teaching method.

**Materials and methods:**

All ninety-nine fifth-year medical students attended a conventional classroom lecture. To teach students the actual procedure, they were randomly divided into two groups: One received conventional verbal explanations and educator demonstrations (the conventional group [*n* = 44]), and the other received VR–IR simulator training (the VR–IR simulator group [*n* = 55]). Afterward, they underwent a test using an augmented reality- (AR-) IR simulator (the VIST^®^ G5 image-guided AR–IR simulator, Mentice, Gothenburg, Sweden). The total procedure time, amount of contrast media used, fluoroscopic time, and patient peak skin dose in the simulated patients were compared between groups. A board-certified radiologist evaluated ten aspects of the procedure technique using a 5-point Likert scale (total: 50 points).

**Results:**

Two students in the VR–IR simulator group were excluded due to VR sickness and simulator malfunction. There were no significant differences between the VR–IR simulator group and the conventional group regarding total procedure time (median [25–75% interquartile range]: 13.5 [11.8–14.5] vs. 14.3 [12.3–16.8] minutes, *p* = 0.11), fluoroscopic time (10.1 [8.5–13.0] vs. 11.0 [8.6–13.7] minutes, *p* = 0.31), and patient peak skin dose (276 [243–373] vs. 303 [239–395] mGy, *p* = 0.57), respectively. However, the amount of contrast media used was significantly lower (28.0 [21.0–36.2] vs. 40.0 [32.3–50.9] mL, *p* < 0.01) and the technical achievement scores by the radiologist (36 [34–44] vs. 31 [29–32], *p* < 0.01) were significantly higher in the VR–IR simulator group.

**Conclusion:**

The VR–IR simulator helped reduce the amount of contrast media in interventional procedures and improved technical achievement scores.

## Introduction

The use of simulation technology for teaching interventional radiology (IR) procedures enables hands-on experience in a controlled environment [1], enhancing efficiency and safety. Augmented reality (AR) enhances real-world scenes, while virtual reality (VR) creates entirely immersive environments. In this context, AR and VR have distinct applications in medical training. Several commercial AR simulators are already being used for IR training [1, 2]. Studies have consistently demonstrated the efficacy of simulators for the education of medical students and novices [3–8]. Before the coronavirus infectious disease (COVID)-19 pandemic, our clinical practice used commercially available AR–IR simulators, alongside physical models and actual medical instruments.

The COVID-19 pandemic severely constrained traditional medical education, necessitating a shift toward web-based lectures to continue training while minimizing the risk of infection. This significantly accelerated the use of technological solutions in education [9–12]. This shift is evident through various innovative approaches that have been reported in the field of radiology education [13–16].

In response to the challenges posed by the pandemic, our diagnostic radiology department re-evaluated its pre-pandemic educational strategies for the medical student education. Previously, we utilized the VIST^®^ G5 simulator (Mentice, Gothenburg, Sweden), a commercial image-guided AR–IR system, which allows the simulation of angiographic procedures using personal computer (PC)-based software and instrument-like interfaces. The simulator was installed in a dedicated training room at our university hospital. However, pandemic-related campus restrictions impeded regular use in medical student education, prompting us to develop alternative educational tools to facilitate remote learning.

We developed a VR-based IR simulator, driven by the notion that VR technologies are particularly well-suited for IR training [17, 18]. This development was also motivated by the belief that active learning through simulation is more effective than traditional lectures or passive observation of procedures. The aim of this study was to introduce the newly developed VR–IR simulator and evaluate its effectiveness for teaching medical students in comparison with our conventional education methods.

## Materials and methods

### Interventional radiology simulator with virtual reality

**Fig. 1:**
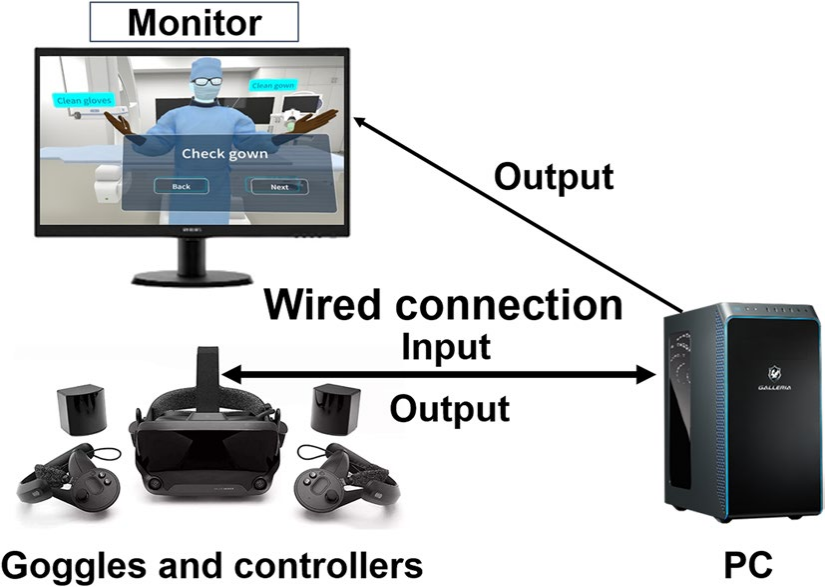
Components of the VR–IR simulator. The learner wears a head-mounted display (goggles and controllers) and learns the procedure virtually while viewing the computer graphic image on the screen. The instructor can share the learner’s actions with others through the projected image on the monitor

We have introduced our newly developed VR–IR simulator which we developed from April 2021 to March 2022. The simulator setup requires each learner to use an individual system comprising a PC, a set of virtual reality goggles, and controllers (Fig. 1). Wearing the goggles, learners view computer-generated images of an angiography room, a patient model, and detailed visuals of abdominal and pelvic vessels. The simulation includes virtual operations such as catheter navigation, transarterial infusion, and embolization of therapeutic agents into tumors or bleeding sites. The system design requires high-speed data processing due to the need for immediate responses from the learner via controllers. Consequently, we tethered a high-performance PC with an advanced graphics board to the goggles to ensure seamless operation. A notebook PC was utilized for portability.

### Educational contents of the virtual reality-interventional radiology simulator

**Fig. 2:**
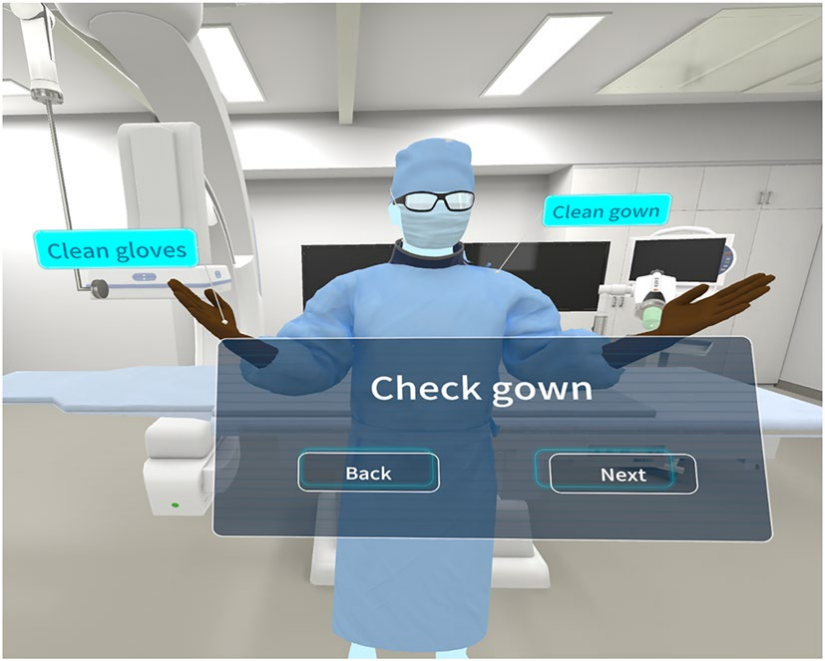
The 3D surgeon VR model in the VR–IR simulator. Students learn how to properly wear radiation-protective clothing, dosimeters, clean gowns, and gloves by observing a properly fitted 3D surgeon model in a VR–IR simulator

**Fig. 3:**
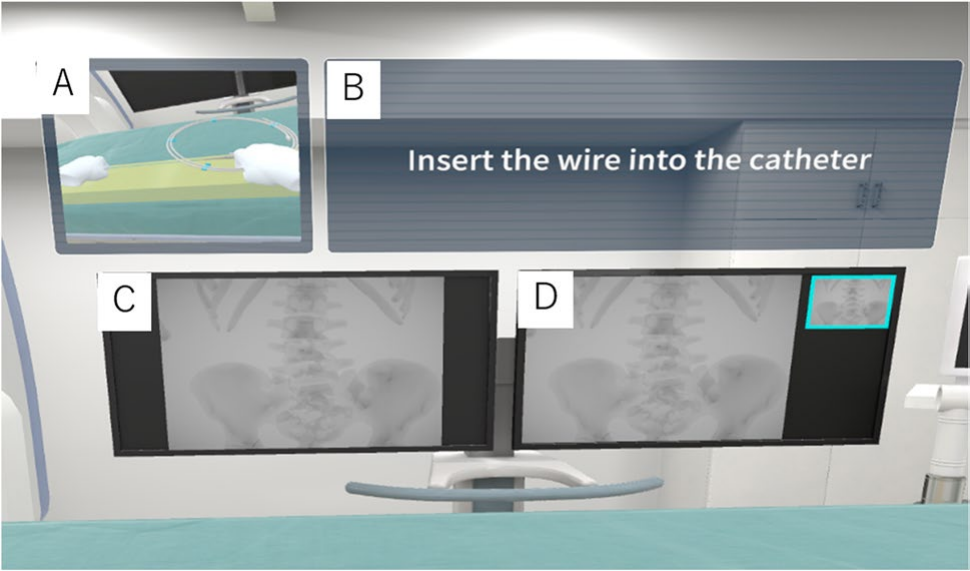
Screengrabs of the instructions with a diagram. A (upper left): The diagram. B (upper right): The text instructions. Instructions with a schematic illustration of the procedure make it easier to understand the location and form of the equipment. C (lower left): A live monitor shows real-time movements under fluoroscopy. D (lower right): A reference monitor

The VR–IR simulator training consists of:

1. *Simulation of protective gear* Learners engage with a 3D virtual model of a surgeon dressed in radiation-protective clothing with dosimeters, sterile gowns and gloves. The simulation focuses on visual recognition of proper attire rather than physically attiring oneself (Fig. 2).
2. *Simulation of cases* The simulator teaches procedures in two anatomical regions: The abdomen and the pelvis. It includes simulations for two cases of transarterial chemoembolization (TACE) for hepatocellular carcinoma (HCC), one hemostatic technique for managing hemorrhage in the abdomen (liver), and three hemostatic techniques in the pelvis. The present study evaluated one TACE case.
3. *Fluoroscopy operation* Trainees practice adjusting the patient bed to align the imaging area under fluoroscopy, which can be activated or deactivated via a controller button. The simulator calculates and displays the virtual radiation dose post-procedure, emphasizing the importance of minimizing fluoroscopy usage to reduce unnecessary exposure.
4. *Catheter and wire techniques* The simulation allows virtual practice of advancing a guidewire, positioning a catheter, and manually injecting contrast media and therapeutic agents using a syringe. Procedures for targeting and embolizing the correct areas are also included.
5. *Self-learning tools* To facilitate self-learning, specific goals of each procedural step are outlined on the screen (Fig. 3). The steps are accompanied by illustrations and text instructions, helping learners to understand the layout and functionality of the equipment.

### Study population and evaluation

Ninety-nine fifth-year medical students who underwent training in TACE for HCC in a 120-min class from April 2022 to March 2023 were included in this study. Training classes were conducted in groups of four to five students. The focus of the fifth year of medical school in Japan is primarily on clinical clerkships, which are similar to clinical rotations in the United States. These represent an early stage of training, in which students gain practical experience in various specialties, such as internal medicine and surgery. This study was reviewed by the institutional review board. Informed consent was obtained from all participants prior to enrolment. Regardless of student participation in this study, we ensured that the class content was equal for all students. We did not collect personal data, such as age or gender, to ensure students felt completely anonymous and their responses were unbiased. All ninety-nine fifth-year medical students attended a conventional classroom lecture. The lecture consisted of basic information on the treatment of HCC (TACE) and how to use the AR–IR simulator for testing (the VIST^®^ G5: image-guided AR–IR simulator, Mentice, Gothenburg, Sweden). It was also used simultaneously with up to three VR–IR simulator goggles. To teach students the actual procedure, they were divided into two groups: one group received conventional verbal explanations and educator demonstrations (a conventional group [*n* = 44]), while the other received training on the VR–IR simulator (a VR–IR simulator group [*n* = 55]). At the beginning of the year, students were divided randomly into groups of four or five according to their student ID numbers. Within each group, students were randomly assigned to the VR–IR simulator group or the conventional group. Some students missed classroom practical sessions due to COVID-19, resulting in unequal numbers in the two groups.

**Fig. 4:**
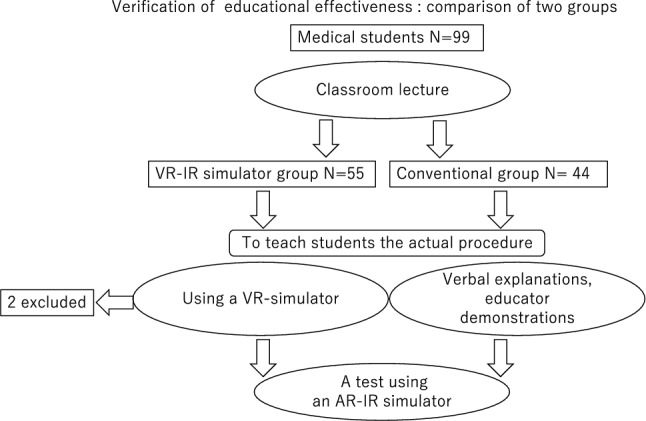
Schematic illustration of the study design. All ninety-nine fifth-year medical students attended a lecture on HCC treatment (TACE) and how to use the AR–IR simulator. To teach students the actual procedure, they were divided into two groups: one received verbal explanations and educator demonstrations (the conventional group, *n* = 44), the other underwent training with the VR–IR simulator (the VR–IR simulator group, *n* = 55). Afterward, they performed a test on the AR–IR simulator simulating an actual patient procedure. Two students from the VR–IR simulator group were excluded due to VR sickness and simulator malfunction

After training, they underwent a test using an AR–IR simulator (Fig. 4). The AR–IR simulator was used as the simulated actual patient for testing. The total procedure time, amount of contrast media used, fluoroscopic time, and patient peak skin dose in the simulated patient were compared between the two groups. Two board-certified interventional radiologists of the Japanese Society of Interventional Radiology (H.M. with 10 years of experience in IR and K.C. with 22 years of experience in IR) created a modified technical achievement score with reference to one previous article to suit TACE procedure [19]. The technical achievement scores, evaluated by one radiologist (K.N., a board-certified diagnostic radiologist of the Japan Radiological Society with 10 years of experience in IR), were also compared between the two groups. The evaluation assessed 10 aspects of the procedure technique (Table 1) using a Likert scale ranging from 1 to 5, with a total score of 50 points.

**Table 1:** Technical achievement score evaluated by the interventional radiologist

	Rate the student on a Likert scale (1–5) for each aspect
1	While the student was moving the wire, was the wire on the fluoroscopy image?
2	Did the student advance catheter over the wire to the aorta?
3	Did the student handle the 4-Fr. catheter adequately?
4	Did the student perform celiac arteriography adequately?
5	Did the student adequately plan the approach to the feeding artery?
6	Did the student adequately handle the microcatheter and the microwire?
7	Did the student reach the feeding artery smoothly?
8	Did the student perform appropriate chemotherapy and embolization?
9	Did the student adequately remove the system after embolization?
10	Throughout the procedure, did the student handle fluoroscopy adequately?

### Statistical analysis

Statistical analysis was performed using R (version 3.2.3). Between-group differences were assessed using the Mann–Whitney* U*-test and *p* < 0.05 was considered indicative of a significant difference.

Data are presented as medians with interquartile ranges [25–75%].

## Results

**Fig. 5:**
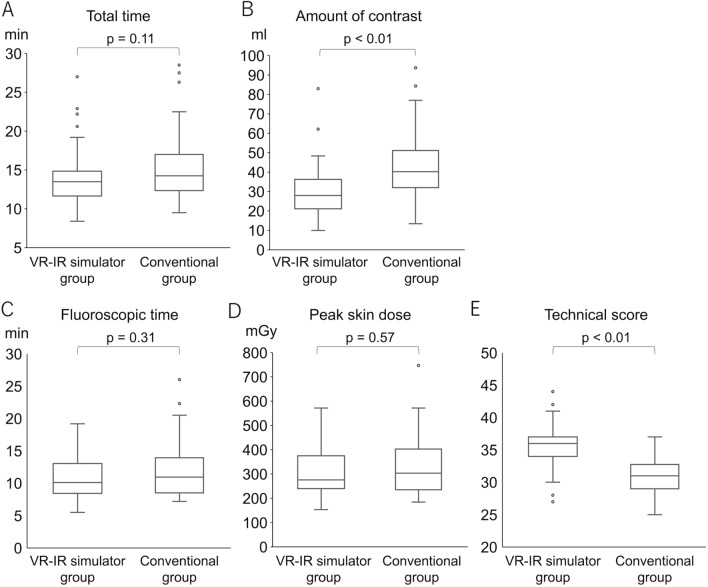
Comparison between the VR–IR simulator group and the conventional group. Results of TACE for HCC in a simulated actual case using AR–IR simulator (VIST^®^ G5) in both groups. **A** The median [25–75% interquartile range] total procedure time was 13.5 [11.8–14.5] minutes in the VR–IR simulator group, whereas 14.3 [12.3–16.8] minutes in the conventional group (*p* = 0.11). **B** The amount of contrast media used was significantly lower at 28.0 [21.0–36.2] mL in the VR–IR simulator group, whereas 40.0 [32.3–50.9] mL in the conventional group (*p* < 0.01). **C** The fluoroscopic time was 10.1 [8.5–13.0] minutes in the VR–IR simulator group, whereas 11.0 [8.6–13.7] minutes in the conventional group (*p* = 0.31). **D** The patient peak skin dose was 276 [243–373] mGy in the VR–IR simulator group, whereas 303 [239–395] mGy in the conventional group (*p* = 0.57). **E** The technical achievement scores by the radiologist were 36 [34–44] in the VR–IR simulator group, whereas 31 [29–32] in the conventional group (*p* < 0.01)

Two students in the VR–IR simulator group were excluded due to VR sickness and simulator malfunction. Finally, we enrolled 44 students in the conventional group and 53 students in the VR–IR simulator group. The results are shown in Fig. 5.

There were no significant differences between the VR–IR simulator group and the conventional group with respect to total procedure time (13.5 [11.8–14.5] vs. 14.3 [12.3–16.8] minutes, *p* = 0.11), fluoroscopic time (10.1 [8.5–13.0] vs. 11.0 [8.6–13.7] minutes, *p* = 0.31), and patient peak skin dose (276 [243–373] vs. 303 [239–395] mGy, *p* = 0.57), respectively. However, in the VR–IR simulator group, the amount of contrast media used was significantly lower (28.0 [21.0–36.2] vs. 40.0 [32.3–50.9] mL, *p* < 0.01) and the technical achievement scores by the board-certified radiologist (36 [34–44] vs. 31 [29–32], *p* < 0.01) were significantly higher than those in the conventional group.

## Discussion

In this study, the group trained with the VR–IR simulator received higher evaluations from the radiologist compared to the conventional group without the VR–IR simulator training. Due to the limited classroom time and the fact that only one AR–IR simulator (VIST^®^ G5) was available for classroom use, whereas three VR goggles could be used simultaneously, a difference in procedural education methods arose between the conventional group and the VR–IR group. Higher evaluations from the radiologist suggest that students in the VR–IR simulator group had a better grasp of procedural steps. These results are consistent with previous studies that support the integration of simulation in IR education to enhance student engagement and proficiency [6, 20]. Stoehr et al. reported similar improvements in student enthusiasm with practical IR simulation training [21]. Early exposure to simulation-based training might influence medical students’ career trajectories, aligning with their preference for technology-driven, autonomous learning environments.

The group trained with the VR–IR simulator used significantly less contrast media compared to the conventional group without the VR–IR simulator training. While the VR–IR simulator group exhibited better median values for procedure time, fluoroscopic time, and patient peak skin dose compared to the conventional group, these differences were not statistically significant. The lack of significant difference in procedure time could be attributed to the efficiency of the AR–IR simulator (VIST^®^ G5), which allows for quick administration of contrast media with the wire in place. Assessment by these items may not be an appropriate indicator of educational effectiveness for medical students using the simulator for the first time in class. Other factors, such as engagement, comfort, and additional survey-based measures, may better capture the real usefulness of the VR–IR simulator in medical student training.

Similar to the present study, various technology-driven initiatives for training medical students and trainees during the COVID-19 pandemic were reported [16]. Kesselman et al. continued safe training using an endovascular simulator during the pandemic [13]. Some researchers have expressed ideas similar to ours about a VR–IR simulator [17, 18], aiming to provide flexible, anytime-anywhere learning resources.

Despite its innovative approach, simulator-based training may not fully meet the self-learning objectives of inexperienced medical students who lack practical exposure and require direct instructor guidance. In the study by Ojala et al., the presence of an instructor was found to facilitate self-directed learning for medical students using VR simulators [22]. However, radiology residents with some IR experience were able to learn independently using the simulator. Thus, instructor guidance seems to be particularly beneficial for beginners or those unfamiliar with the operations after which self-directed learning can be effective for continuing education.

Despite several advantages of the VR–IR simulator, some limitations must be considered. First, only a limited number of case scenarios are available for study on the simulator. Ideally, real-world case data would be converted into simulator-ready formats for pre-procedural practice, but this was not achievable. Second, due to VR sickness and equipment malfunction, two participants could not receive the education. Third, the development and maintenance of VR–IR simulators is a costly endeavor. Future technological advances may reduce these costs, malfunctions, and side effects. Fourth, our VR–IR simulator does not replicate the tactile sensations of touch and pressure, which play a crucial role in real-world catheter and wire techniques. Although this sensory feedback is essential for experienced specialists, we focused on visual and procedural training for novice students, considering it an effective initial learning strategy. Fifth, information on the group was not blinded during evaluation of the procedure technique using a score by a radiologist, which may have been a bias during evaluation. Sixth, population characteristics (such as age, sex, medical knowledge about TACE, etc.) were not compared between the two groups. Therefore, there may have been a bias that could have affected the results. Lastly, a single radiologist evaluated our original modified technical achievement score, which may have introduced an element of bias.

Despite the challenges, the role of VR in medical education is poised to expand significantly, driven by ongoing technological advancements. Beyond educational applications, VR is expected to extend into clinical practice, including preoperative simulations.

## Conclusion

We developed a VR–IR simulator and demonstrated its effectiveness for training medical students. The VR–IR simulator was found to help reduce the amount of contrast media used in IR procedures and to improve the technical achievement scores of medical students.

## Abbreviations

IR: Interventional radiology
VR: Virtual reality
VR–IR simulator: Virtual reality–interventional radiology simulator
AR: Augmented reality
AR–IR simulator: Augmented reality–interventional radiology simulator
COVID: Coronavirus infectious disease
PC: Personal computer
TACE: Transarterial chemoembolization
HCC: Hepatocellular carcinoma
